# The relationship between working condition factors and well-being

**DOI:** 10.1186/s40557-014-0034-z

**Published:** 2014-11-04

**Authors:** Bum-Joon Lee, Shin-Goo Park, Kyoung-Bok Min, Jin-Young Min, Sang-Hee Hwang, Jong-Han Leem, Hwan-Cheol Kim, Sung-Hwan Jeon, Yong-Seok Heo, So-Hyun Moon

**Affiliations:** 1 Department of Occupational and Environmental Medicine, Inha University, 7-206. 3-GA, Sinheung-dong, Jung-Gu, Incheon, Republic of Korea; 2 Department of Dentistry, Keimyung University School of Medicine, Daegu, Republic of Korea; 3 Department of Occupational & Environmental Medicine, Ajou University School of Medicine, Suwon, Republic of Korea; 4 Institute of Health and Environment, Seoul National University, Seoul, Republic of Korea

**Keywords:** Working condition survey, Employed worker, Working conditions, Well-being

## Abstract

**Objectives:**

Working conditions can exert influence on the physical, mental, and even social health of workers. Well-being is an appropriate index for the evaluation of a person’s overall health. This paper investigated the association between various working conditions and worker’s well-being.

**Methods:**

Data from 10,019 interviews were collected from the second wave of the Korean Working Conditions Survey (2010) conducted by the Korea Occupational Safety and Health Agency between June and October 2010. The data from 5,995 employed workers were examined in this study. Well-being was measured through the WHO Five Well-Being Index (1998 version). Sociodemographic and working conditions were analyzed. Adjusted odds ratios for well-being were calculated with adjusted sociodemographic factors, working condition factors, or both.

**Results:**

Workers’ well-being was significantly higher when they were satisfied with their working conditions (OR = 1.656, 95% CI = 1.454–1.885), when their actual working hours were the same as their anticipated working hours (OR = 1.366, 95% CI: 1.120–1.666) or exceeding less than 10 hours (OR = 1.245, 95% CI: 1.004-1.543), and when their employment was stable (OR = 1.269, 95% CI: 1.098–1.467).

**Conclusions:**

This study supports the association between working condition factors and well-being in workers.

## Introduction

Working conditions have significant influence on a worker’s quality of life. Workers spend more than a half of their waking hours on working [1]. Therefore, naturally, working conditions may exert influence on other areas of life, including eating, sleeping, housing, and developing interpersonal relationships, directly or indirectly. Moreover, Working conditions affect workers’ physical and mental health [2]. Due to industrial development, working conditions have become more diverse and complicated. Therefore, it is essential to closely measure and evaluate the influence of each working condition on workers’ health as society continues to evolve and change.

As industry has developed and the problems presented by poverty and illness have been resolved, society’s priority has transitioned from “survival” to “better life”. “The measurement for well-being (such as WHO well-being index)” is an appropriate measurement of a subject’s physical, mental, and social health because it encompasses the absence of both illness and negative emotions [3]. Well-being so closely related to mental health that subjects with a low WHO Five well-being index score are often recommended for depression screening tests [4]. Well-being has been linked with physical health by several studies that demonstrate that satisfied individuals have stronger immune systems and enjoy better physical health [5]-[7]. Additionally, there seems to be a positive feedback loop between a worker’s well-being and productivity. Individuals with high well-being are more productive in occupational activities than are individuals with low well-being, and productive individuals are happier than non-productive individuals [8].

Many previous studies on the influence of working conditions on health have focused on the occurrence of specific diseases like musculoskeletal illness [9],[10], occupational cancers [11], and sleep disorders [12],[13] in risky working conditions, or the influence of job stresses, job satisfaction on life habits like smoking and drinking [14],[15]. Although a few studies examined the influence of psychological occupational factors on well-being in specific occupations like teaching [16], there is a lack of research on the influence of overall working conditions on well-being. The present study examines how the working conditions are related to employed workers’ well-being. Data were gathered from the second wave of the Korean Working Conditions Survey.

## Materials and methods

### Working conditions survey

This study used a sample from the second wave of the Korean Working Conditions Survey (2010) conducted by the Korea Occupational Safety and Health Agency. The methodology and survey questionnaire used for the second Korean Working Conditions Survey is similar to those used in the European Working Conditions Survey, and the second survey built on investigations begun in the first survey (2006).

Briefly, the goal of the survey was to gather comprehensive information on Korean working conditions to shed light the nature and types of changes affecting the workforce and the quality of work-life for employees. The specific objective of the survey was to develop social and occupational health indicators for the working environment. We used multi-stage, random sampling from the Enumeration Districts in the 2005 Population and Housing Census. The survey was carried out at a number of different sampling areas, determined using probability proportional to population size and to population density. The survey data were collected from a nationally representative sample of the economically active population 15 years and older, excluding retirees, unemployed, homemakers, and students [17].

In this study, we defined the subjects as only ‘employed workers’, so we exclude ‘a self-employed worker’ or ‘an unpaid worker for familial business. With these exclusion criteria, among 10,019 survey interviews conducted at workers' homes between June and October 2010, the data from only 5,995 employed workers were used in this study.

The Institutional Review Board of Inha University Hospital approved the study protocol.

### WHO five well-being index

Well-being was evaluated through the WHO Five Well-Being Index (the 1998 version). Although the index was originally designed to measure well-being in diabetic patients [18], its effectiveness has been supported in diagnostic depression screening [4] and evaluation of emotional well-being in patients with chronic diseases including cardiovascular diseases [18] and Parkinson’s disease [19], and in young children [20], and elderly adults [21].

The index consists of five positively worded items, each of which reflects the presence or absence of well-being. Subjects respond to questions about their positive feelings within the last two weeks on 6-point scale (0–5). A raw score lower than 13 out of 30 or an individual item score of 0 or 1 on any of the five items implies poor well-being [22], and a raw point score considerably below 13 may necessitate screening for depression with the Major Depression Inventory (under ICD-10) [4]. This study has evaluated the states of well-being of the subjects by classifying subjects with a raw score below 13 or a score of 0 or 1 on one of more items into the “poor well-being” group. Meanwhile, those who responded to all the items with a score of 2 or higher and had a total score higher than 13 were assigned to the “fair well-being” group [22].

### Definition of independent variables

The survey collected information on several sociodemographic factors. Age, educational levels and monthly income were classified. Based on survey responses, the degrees of difficulty balancing income and expenses with total monthly earnings were divided into “difficult,” “somewhat difficult,” “somewhat easy,” and “easy.” Overall subjective health status, smoking status and alcohol assumption scale were also remarked. Subjects were grouped into “non-drinkers,” “moderate drinkers,” and “excessive drinkers” (i.e., individuals who consumed more than 2 glasses of alcohol a day).

Information on working condition factors was also collected and organized. Occupations were divided into 9 categories based on the Korean employment classification of occupation. Length of employment was divided into “<1 year,” “1–5 years,” “5–10 years,” “10+ years.” Employment types were divided into “contingent” and “regular” employees, and employment stability (“stable” or “unstable”) was determined by researchers based on the subjects’ responses. The presence and the absence of shifts were noted, and total weekly work hours were categorized into “<40 hours,” “40 hours,” ”40–60 hours,” and “60+ hours.” Based on the calculated difference between the actual and desired working time, we had categories of individuals who “lacked work time,” worked “10+ hours longer” than desired, worked “<10 hours longer” than desired or experienced “no difference” between actual and desired work time. Monthly work on the weekends was divided into “4+ days a month,” “1–4 days,” and “none.” Work condition satisfaction was divided into “satisfied” and “unsatisfied.” Degree of work stress was divided into the categories of “mild,” “moderate,” and “severe.”

### Statistical analysis

All data were analyzed with the SPSS (version 14.0) after encoding was completed. A descriptive analysis was carried out on sociodemographic and working condition factors. Pearson’s chi-squared analysis was carried out between each factor and well-being. Logistic regressions yielded crude and adjusted odds ratios of working condition factors and the well-being. For logistic regression analysis, three models were used for adjusting effect of other factors. The model 1 was adjusted for sociodemographic factors (gender, age, education, monthly income, balancing income and expenses, health status, smoking, and drinking); the model 2 was adjusted for working conditional factors (type of occupation, number of years employed, employment type, employment stability, weekly working time, difference between actual and desired working time, monthly weekend work, satisfaction with working conditions, and work stress); and the model 3 was adjusted for both sociodemographic and working conditional factors. Pearson correlation analysis was used to test for multicollinearity among individual factors. The significance threshold was 0.05.

## Results

### Sociodemographic factors and well-being

The average raw score of the WHO Five Well-being Index in the 5,995 participants was 13.29 (SD: 5.53); 2,415 (40.3%) were in the poor well-being group, while 3,580 (59.7%) were in the fair well-being group.

Well-being did not significantly differ between genders. Subjects who were under 30 or 40–49 years old had higher well-being scores. Subjects with higher education levels had significantly higher scores of well-being. Subjects with income greater than 3 million KRW had significantly higher well-being than those with lower incomes. Subjects who experienced a balance between their income and expenses (income-expense balance) had significantly higher well-being than those who faced an imbalance between income and expenses. Workers who reported better health status had significantly higher scores of well-being. Smoking and drinking were both related to well-being (Table 1).

**Table 1:** Sociodemographic factors and well-being

			**Well-being**				
		**Total**	**Poor**		**Fair**		
		**N**	**N**	**%**	**N**	**%**	p-value*
Total		5,995	2,415	40.3	3,580	59.7	
Gender	Male	3,304	1,341	40.6	1,963	59.4	0.595
	Female	2,691	1,074	39.9	1,617	60.1	
Age	<29	901	328	36.4	573	63.6	<0.001
	30–39	1,833	790	43.1	1,043	56.9	
	40–49	1,662	602	36.2	1,060	63.8	
	50–59	1,018	426	41.8	592	58.2	
	>60	581	269	46.3	312	53.7	
Education	Middle school or lower	917	455	49.6	462	50.4	<0.001
	High school	2,578	1,057	41.0	1,521	59.0	
	Junior college	755	325	43.0	430	57.0	
	College or higher	1,745	578	33.1	1,167	66.9	
Monthly income (KRW)	<1 million	1,326	572	43.1	754	56.9	<0.001
	1≤, <2 million	2,251	939	41.7	1,312	58.3	
	2≤, <3 million	1,382	547	39.6	835	60.4	
	≥3 million	1,036	357	34.5	679	65.5	
Balancing income and expenses	Difficult	1,149	669	58.2	480	41.8	<0.001
	Somewhat difficult	2,122	986	46.5	1,136	53.5	
	Somewhat easy	1,740	525	30.2	1,215	69.8	
	Easy	984	235	23.9	749	76.1	
Health status	Bad	255	171	67.1	84	32.9	<0.001
	Normal	1,396	831	59.5	565	40.5	
	Good	3,572	1,253	35.1	2,319	64.9	
	Very good	772	160	20.7	612	79.3	
Smoking	Non smoker	3,420	1,328	38.8	2,092	61.2	0.025
	Current smoker	1,999	850	42.5	1,149	57.5	
	Ex-smoker	576	237	41.1	339	58.9	
Drinking	Non-drinker	1,715	688	40.1	1,027	59.9	<0.001
	Moderate drinker	2,574	976	37.9	1,598	62.1	
	Excessive drinker	1,706	751	44.0	955	56.0	

*p-value: Pearson’s chi-squared test.

### Working condition factors and well-being

A univariate analysis revealed that of the 9 types of jobs, clerks had higher well-being, whereas subjects working in agriculture, forestry and fishing had lower well-being. Workers with 5–10 years’ experience showed somewhat higher well-being than workers with other lengths of work experience in univariate analysis, all the other working condition factors, employment types, employment stability, weekly working time, difference between the actual and desired working time, monthly weekend work, working condition satisfaction, work stress, had statistically significant relationship with workers' subjective well being except shift working (Table 2).

**Table 2:** Working condition factors and well-being

			**Well-being**				
		**Total**	**Poor**		**Fair**		
		**N**	**N**	**%**	**N**	**%**	p-value*
Type of occupation	Unskilled	1,143	531	46.5	612	53.5	<0.001
	Semi-skilled	445	195	43.8	250	56.2	
	Skilled	685	300	43.8	385	56.2	
	Expertise	492	175	35.6	317	64.4	
	Sales	727	278	38.2	449	61.8	
	Service	922	402	43.6	520	56.4	
	Clerk	1,494	492	32.9	1,002	67.1	
	Senior management	53	18	34.0	35	66.0	
	Agriculture, forestry and fishing	34	24	70.6	10	29.4	
Years employed	<1 yr	1,166	486	41.7	680	58.3	0.023
	1≤, <5 yrs	2,847	1,179	41.4	1,668	58.6	
	5≤, ≤10 yrs	967	350	36.2	617	63.8	
	>10 yrs	1,015	400	39.4	615	60.6	
Employment type	Contingent	1,288	601	46.7	687	53.3	<0.001
	Regular	4,707	1,814	38.5	2,893	61.5	
Employment stability	Unstable	1,871	830	44.4	1,041	55.6	<0.001
	Stable	4,124	1,585	38.4	2,539	61.6	
Shift working	Present	605	243	40.2	362	59.8	0.950
	Absent	5,390	2,172	40.3	3,218	59.7	
Weekly working time	<40 hrs	929	388	41.8	541	58.2	<0.001
	40 hrs	1,893	648	34.2	1,245	65.8	
	40<, ≤60 hrs	2,687	1,142	42.5	1,545	57.5	
	>60 hrs	486	237	48.8	249	51.2	
Difference between the actual and desired working time	≥10 hrs longer	774	395	51.0	379	49.0	<0.001
	<10 hrs longer	1,128	461	40.9	667	59.1	
	No difference	3,390	1,240	36.6	2,150	63.4	
	Lack of working time	703	319	45.4	384	54.6	
Monthly weekend work	More than 4 days	835	368	44.1	467	55.9	<0.001
	1 ~ 4 days	2,611	1,166	44.7	1,445	55.3	
	none	2,549	881	34.6	1,668	65.4	
Working condition satisfaction	Dissatisfied	1,795	1,004	55.9	791	44.1	<0.001
	Satisfied	4,200	1,411	33.6	2,789	66.4	
Work stress	Severe	1,855	789	42.5	1,066	57.5	0.018
	Moderate	2,472	995	40.3	1,477	59.7	
	Mild	1,668	631	37.8	1,037	62.2	

*p-value: Pearson’s chi-squared test.

In the analysis of the 9 types of jobs, only the model 2 showed a significant difference with an OR of 1.251 in office workers (95% CI: 1.042–1.502), and an OR of 0.433 in farmers and fishermen (95% CI: 0.200–0.937), but the difference was not significant in model 3. No relationship was observed between employment types and well-being in model 1, 2 and 3. A significant relationship between employment stability and well-being was present. Well-being was significantly higher when employment was stable, and this pattern remained constant in all the 3 models (model 1: OR = 1.204, 95% CI: 1.063–1.365; model 2: OR = 1.222, 95% CI: 1.065–1.402; model 3: OR = 1.269, 95% CI: 1.098–1.467).

A relationship was found between well-being and the difference between workers’ actual and desired working time. Subjects who reported that their actual working hours were as desired or exceeding less than 10 hours showed significantly higher well-being than subjects who worked ≥10 hours longer than desired. The OR was 1.533 (95% CI: 1.294–1.816), 1.297 (95% CI: 1.065-1.580) in model 1, 1.401 (95% CI: 1.159–1.693), 1.305 (95% CI: 1.064-1.602) in model 2, and 1.366 (95% CI: 1.120–1.666), 1.245 (95% CI: 1.004-1.543) in model 3. Additionally, subjects who did not work on weekends showed higher well-being than those who worked 4+ weekend days a month. These results were constant only in model 1.

Satisfaction or non-satisfaction with working conditions was related to well-being. In all the three models, subjects who were satisfied with their working conditions demonstrated significantly higher well-being. (Model 1: OR = 1.723, 95% CI: 1.519–1.955; Model 2: OR = 2.209, 95% CI: 1.957–2.492; Model 3: OR = 1.656, 95% CI: 1.454–1.885).

After adjusted for sociodemographic or working conditional factor, unremarkable relationship was found between degree of job stress and well-being (Table 3).

**Table 3:** Working condition factors and well being (Logistic regression analysis)

		**Crude OR**		**Model 1**		**Model 2**		**Model 3**	
		**OR**	**95% CI**	**OR**	**95% CI**	**OR**	**95% CI**	**OR**	**95% CI**
Type of occupation	Unskilled	1.000	-	1.000	-	1.000	-	1.000	-
	Semi-skilled	1.112	0.892-1.387	1.183	0.924-1.515	1.194	0.946 -1.506	1.198	0.932 -1.540
	Skilled	1.113	0.920-1.347	1.032	0.817-1.303	1.046	0.851 -1.286	0.996	0.785 -1.264
	Expertise	1.572	1.264-1.955	0.974	0.731-1.298	1.192	0.942 -1.507	0.927	0.691 -1.243
	Sales	1.401	1.160-1.694	1.008	0.804-1.262	1.206	0.982 -1.481	0.980	0.777 -1.236
	Service	1.122	0.943-1.336	0.910	0.744-1.114	1.067	0.885 -1.286	0.933	0.758 -1.148
	Clerk	1.767	1.508-2.071	1.203	0.963-1.503	1.251	1.042 -1.502	1.043	0.828 -1.314
	Senior management	1.687	0.944-3.014	1.086	0.571-2.068	1.167	0.640 -2.128	0.956	0.499 -1.834
	Agriculture, forestry and fishing	0.362	0.171-0.763	0.430	0.195-0.947	0.433	0.200 -0.937	0.468	0.209 -1.048
Years employed	<1 yr	1.000	-	1.000	-	1.000	-	1.000	-
	1≤, <5 yrs	1.011	0.881-1.161	0.991	0.849-1.158	0.931	0.802 -1.081	0.970	0.827 -1.138
	5≤, ≤10 yrs	1.260	1.057-1.501	1.271	1.038-1.557	1.079	0.892 -1.307	1.180	0.957 -1.454
	>10 yrs	1.099	0.926-1.304	1.083	0.871-1.347	0.862	0.711 -1.046	0.972	0.775 -1.220
Employment type	Contingent	1.000	-	1.000	-	1.000	-	1.000	-
	Regular	1.395	1.232-1.580	1.098	0.949-1.271	1.112	0.950 -1.303	0.982	0.828 -1.164
Employment stability	Unstable	1.000	-	1.000	-	1.000	-	1.000	-
	Stable	1.277	1.143-1.427	1.204	1.063-1.365	1.222	1.065 -1.402	1.269	1.098 -1.467
Weekly working time	<40 hrs	1.000	-	1.000	-	1.000	-	1.000	-
	40 hrs	1.378	1.173-1.619	1.067	0.871-1.306	0.975	0.795 -1.195	0.971	0.773 -1.219
	40<, ≤60 hrs	0.970	0.834-1.129	0.861	0.709-1.046	0.922	0.749 -1.134	0.962	0.760 -1.218
	>60 hrs	0.754	0.604-0.939	0.773	0.596-1.002	0.962	0.723 -1.279	1.040	0.758 -1.427
Difference between the actual and desired working time	≥10 hrs longer	1.000	-	1.000	-	1.000	-	1.000	-
	<10 hrs longer	1.508	1.254-1.813	1.297	1.065-1.580	1.305	1.064 -1.602	1.245	1.004 -1.543
	No difference	1.807	1.544-2.115	1.533	1.294-1.816	1.401	1.159 -1.693	1.366	1.120 -1.666
	Lack of working time	1.255	1.022-1.540	1.444	1.148-1.817	1.142	0.879 -1.483	1.283	0.975 -1.688
Monthly weekend work	1–4 days	1.000	-	1.000	-	1.000	-	1.000	-
	None	0.977	0.835-1.142	0.953	0.804-1.130	0.858	0.726 -1.013	0.887	0.744 -1.058
	>4 days	1.492	1.272-1.749	1.328	1.115-1.583	1.118	0.928 -1.347	1.135	0.932 -1.383
Satisfaction with working conditions	Dissatisfied	1.000	-	1.000	-	1.000	-	1.000	-
	Satisfied	2.509	2.241-2.809	1.723	1.519-1.955	2.209	1.957 -2.492	1.656	1.454 -1.885
Work stress	Severe	1.000	-	1.000	-	1.000	-	1.000	-
	Moderate	1.099	0.972-1.242	1.051	0.921-1.199	1.005	0.885 -1.142	0.990	0.865 -1.133
	Mild	1.216	1.063-1.392	1.144	0.987-1.327	1.112	0.963 -1.284	1.031	0.885 -1.201

Model 1: Adjusted for sociodemographic factors.

Model 2: Adjusted for working conditional factors.

Model 3: Adjusted for both sociodemographic and working conditional factors.

## Discussion

We conducted this study to evaluate the association between general working condition factors and employed workers' well-being in a nationwide representative sample of Korea. After we adjusted sociodemographic and working condition factors, workers’ well-being was significantly higher when they were satisfied with their working conditions (OR = 1.656, 95% CI: 1.454–1.885), when their actual working hours were the same as their desired working hours (OR = 1.366, 95% CI: 1.120–1.666) or exceeding less than 10 hours (OR = 1.245, 95% CI: 1.004-1.543), and when their employment was stable (OR = 1.269, 95% CI: 1.098–1.467).

This current study is the first one that studied the relationship between general working conditional factors and workers’ well-being. Therefore, few comparable outcomes exist. Previous findings [23] merely compared a few working conditions such as employees to the unemployed, and professionals to inexperienced workers. Previous studies have not addressed the relationship between other working conditional factors and well-being.

The sociodemographic factors were drawn from a nationwide survey on working conditions and included gender, age, educational level, monthly income, income-expense balance, health status, smoking, and drinking. The workers’ well-being has no differences between the genders. This finding is consistent with a previous study that gender accounts little for the variance in individual differences in well-being [24]. Older subjects tended to have lower well-being. This result contradicts the finding that older individuals are happier with their lives than younger individuals [25]. However, another study [26] proposed that older individuals tended to have lower well-being than younger individuals because the former experience more health problems and less satisfaction with the future than the latter. That study and the current one both found a positive correlation between education levels and well-being. Another research [27] has suggested a correlation between income and well-being at lower income levels but not at higher income levels. In the current study, the subjects with a monthly income of 3 million or more KRW had significantly higher well-being than subjects with a lower income. However, income-expense balance had a stronger correlation with well-being than absolute monthly income did. Subjects who reported it was either somewhat difficult or easy to balance their income and expenses experienced higher well-being than those who reported it was difficult.

Subjects with normal, good, or very good self-reported health status had higher well-being than those with poor self-reported health status. This finding accords with the results of a previous study [23]. Interestingly, only 264 subjects (4.2%) had bad self-reported health status, whereas 4,571 (72.6%) reported their health status was “good” and “very good.” We can hypothesize that there is a “healthy worker effect” that may occur in surveys that are distributed only to workers who are currently employed.

Current smokers had somewhat lower scores of well-being than non-smokers, respectively. Moderate drinkers showed higher well-being status than non-drinkers. Excessive drinkers showed the lowest well-being status. This result is consistent with previous findings [28]-[30] that suggest that smoking and excessive drinking are detrimental to physical as well as mental health, as measured by life satisfaction and well-being.

Fifth EWCS (2010) evaluated mental well-being of the workers by using WHO-Five well-being index. According to the fifth EWCS Overview report (2012), the result of the index regarded as a good predictor of mortality and functional ability. 20% (male: 18%, female: 22%) of the workers of the EU 27 countries were classified into 'mental health at risk'. Percentage of the elder workers (above 50 year-old) was 7 percent point more than the younger's (below 35 year-old). In this study, the percentage of low mental well-being group in Korean employed workers was 40.3%. Comparing between this study and fifth EWCS Overview report, well-being of the Korean employed workers were lower than average of the 27 EU counties. The relations between ages and well-being or job and well-being were similar. There are little information about the relation between well-being and working conditional factors in EWCS report [31].

This study, however, has several limitations. First, although it demonstrates a relationship between working condition factors and well-being, we cannot make conclusions regarding causality. Second, we did not take into account the “healthy worker effect” during our analysis, which could influence our results. Third, we did not examine variations in individual personality traits that could influence workers’ well-being. Although an existing study explored the hypothesis that individuals’ positive personalities are closely related to their well-being [32], we were not able to investigate personality traits because the working conditions survey did not contain the necessary items. Fourth, we didn’t assessed the relationship between well-being and physically or chemically hazardous working conditional factors which could be exist on specific job such as noise, heavy metals, organic chemicals. However, some of these factors could be included in type of occupation.

## Conclusions

This is the first study that analyzed association between general working conditions and workers’ well-being. We expect further research to figure out causal relationships between general or specific working conditions and workers’ well-being.

## Competing interests

The authors declare that they have no competing interests.

## Authors’ contributions

BJ: The first author of this article. He designed the study, interpreted the data, and drafted the manuscript. SG: The corresponding author of this article. He suggested the study design, interpreted the data, and revised the manuscript. KB: He consulted the study method and revised the manuscript. JY: she revised the manuscript. SHH: she translated the manuscript. JH: He suggested the study design and revised the manuscript. HC: He suggested the study design and revised the manuscript. SHJ: He interpreted the data and revised the manuscript. YS: He interpreted the data and revised the manuscript. SHM: She drafted the manuscript. All authors have approved the final version of the manuscript.
